# Cell death-related gene signatures as dual-function biomarkers: Early diagnosis and therapeutic targeting in *Staphylococcus aureus* pneumonia

**DOI:** 10.1371/journal.pone.0339560

**Published:** 2026-01-20

**Authors:** Cao Qing, Wanjuan Sun, Chaomian Yang, Yien Yao, Qiong Liang, Tianxia Huang, Lu Lin

**Affiliations:** 1 Department of Pulmonary and Critical Care Medicine, The First People’s Hospital of Nanning, Nanning, China; 2 Department of Pulmonary and Critical Care Medicine, The Fifth Affiliated Hospital of Guangxi Medical University, Nanning, China; Southern Medical University Nanfang Hospital, CHINA

## Abstract

**Background:**

*Staphylococcus aureus* (*S. aureus*) pneumonia constitutes a lethal respiratory infection with persistently high clinical mortality. Although programmed cell death (PCD) pathways are implicated in diverse disease processes, their mechanistic roles in *S. aureus* pneumonia pathogenesis, particularly as dual-purpose biomarkers for early diagnosis and therapeutic targeting remain insufficiently characterized.

**Methods:**

High-throughput RNA sequencing was conducted on *S. aureus* challenged murine pulmonary tissues to delineate pneumonia-associated differentially expressed genes (DEGs). Through bioinformatics screening, we established a PCD -related gene signature and validated its clinical relevance via transcriptomic profiling of peripheral blood samples from confirmed *S. aureus* pneumonia patients. Machine learning (including LASSO regression and SVM-RFE algorithms) were employed to prioritize characteristic biomarkers, followed by construction of a risk-prediction nomogram with Receiver Operating Characteristic (ROC) curve validation. Multidimensional analyses encompassing immune cell infiltration patterns and DSigDB based drug discovery were performed, supplemented by molecular docking simulations and qPCR confirmation of core regulatory elements.

**Results:**

Whole-transcriptome analysis revealed 71 PCD-related DEGs (DE-PCDs) with conserved cross-species dysregulation (19 genes in human specimens). Machine learning identified 11 hub genes modulating apoptosis, necroptosis, autophagy, and ferroptosis interconnections. A four-gene diagnostic panel (NAMPT, NFKBIA, SLC40A1, PRKCQ) demonstrated high predictive accuracy (AUC = 0.92) via nomogram modeling. Significant correlations emerged between biomarkers and neutrophil/T-cell infiltration dynamics, while computational drug screening identified 27 candidate compounds targeting these determinants.

**Conclusion:**

This investigation delineates PCD-mediated regulatory networks in *S. aureus* pneumonia, establishing a clinically translatable biomarker panel with theranostic potential against infectious pulmonary inflammation.

## Introduction

*Staphylococcus aureus* asymptomatically colonizes the upper respiratory tract in 30% of healthy individuals, yet paradoxically represents a leading cause of pneumonia-associated mortality, particularly in immunocompromised populations [1,2]. Epidemiological data from 2020 indicate 1.5 million emergency department visits and 41,309 deaths attributed to *S. aureus* pneumonia [3]. Recent surveillance further reveals its predominance in ventilator-associated infections, accounting for 22% of such cases [4]. Current diagnostic and therapeutic approaches face dual challenges: the rising prevalence of antibiotic resistance (e.g., methicillin, penicillin, and derivatives) in *S. aureus* isolates, and the pathogen’s ability to trigger dysregulated immune responses characterized by excessive inflammation and tissue damage during pneumonia [5]. Diagnosis is frequently complicated by concurrent comorbidities, and treatment initiation typically occurs only after clinical symptoms (e.g., fever, leukocytosis, and radiographic infiltrates) manifest, resulting in delayed therapeutic intervention. Consequently, the substantial economic burden stems from prolonged hospitalization and intensive care requirements, which strain healthcare resources [6,7]. This therapeutic impasse underscores the urgent need to elucidate host-pathogen interactions governing pulmonary immunopathology, particularly inflammatory cell death pathways that drive tissue injury and may offer dual diagnostic and therapeutic targets.

The pathogenesis of S. aureus pneumonia initiates with microbial aspiration from nasopharyngeal colonization sites, ultimately progressing to destructive lower respiratory tract infections. Programmed cell death, a critical component of immune defense, plays dual roles in bacterial infections, balancing tissue homeostasis through regulated clearance of compromised cells and dynamic responses to pathogens [8–11]. Specifically, distinct PCD pathways, including apoptosis (caspase-mediated), necroptosis (RIPK3/MLKL-driven), NETosis (neutrophil extracellular trap release), and ferroptosis (iron-dependent peroxidation) orchestrate immunopathology via innate immune signaling networks [12].

During infection, immunomodulatory pathways coordinate PCD to restrict intracellular pathogen replication, recruit inflammatory cells, sequester pathogens within dead cells, or release antimicrobial factors like neutrophil extracellular traps (NETs) [12,13]. However, the synergistic crosstalk between PCD pathways and their collective impact on *S. aureus* pneumonia progression remains poorly defined. Recent systematic profiling of host cell death pathways and regulatory networks in *S. aureus* pneumonia has revealed critical insights into pulmonary immune dysregulation [14]. Yet Current studies focus predominantly on isolated death mechanisms, neglecting combined effects on disease severity. In this study, we employ integrated cross-species transcriptomics to map coordinated PCD activation, coupled with machine learning modeling to quantify dynamic pathway interactions and identify critical transition points. Furthermore, we validate a multiplexed PCD biomarker panel for clinical translation. This approach advances targeted diagnostic and therapeutic strategies for pulmonary immunopathology.

Here, we employ whole-transcriptome RNA sequencing coupled with machine learning to decode PCD-mediated immunopathology in *S. aureus* pneumonia. Through integrative analysis of murine lung and human blood transcriptomes, we identify conserved PCD regulators correlating with disease progression. Our computational-experimental pipeline prioritizes four biomarkers (NAMPT, NFKBIA, SLC40A1, PRKCQ) that stratify pneumonia risk and predict targeted drugs for therapeutic repurposing. These findings establish a theranostic strategy targeting PCD-immune crosstalk, providing actionable insights for developing host-directed adjunctive therapies against antibiotic-resistant *S. aureus* pneumonia.

## Materials and methods

### Establishment of *S. aureus* pneumonia murine model

Male C57BL/6 mice (age 4–6 weeks; body weight 18–22 g) were procured from the Specific Pathogen-Free (SPF) facility at Guangxi Medical University Laboratory Animal Center. All experimental protocols were approved by the Institutional Animal Care and Use Committee of Guangxi Medical University (Ethics Approval No. 202412004) in strict compliance with the Guidelines for the Humane Treatment of Laboratory Animals promulgated by the Chinese Ministry of Science and Technology. Following one-week acclimatization under controlled environmental conditions (22 ± 1°C, 55 ± 5% humidity, 12h light/dark cycle), mice were randomized into two cohorts: (1) Phosphate-buffered saline (PBS) control group (n = 10), and (2) *S. aureus* challenge group (n = 10) receiving 50 μL intranasal instillation of methicillin-sensitive *S. aureus* (MSSA) strain ATCC 29213 suspension (1 × 10⁸ CFU/mL in sterile PBS). Prior to bacterial inoculation, deep anesthesia was achieved through intraperitoneal administration of sodium pentobarbital (50 mg/kg body weight). Subsequently, a nasal drip containing a suspension of *S. aureus* at a concentration of 1 × 10^8 CFU was administered. Post-infection (24h), euthanasia was performed with pentobarbital (100 mg/kg, IP) with death verification via apnea≥5 minutes and fixed pupils. Suffering was alleviated through minimized sample size, twice-daily monitoring of behavior/feeding, optimized housing (22°C, 55% humidity), and rapid tissue processing (<15 min). Lung tissues were collected and stored at 4°C. The efficacy of the *S. aureus* infection model was confirmed through hematoxylin and eosin (HE) staining.

### Transcriptome sequencing of lung tissue

Lung tissues were harvested for high-throughput mRNA sequencing. For each RNA sample, 1 μg of RNA was utilized as the starting material. Eukaryotic mRNA sequencing was conducted using the Illumina NovaSeq platform, employing the Illumina NovaSeq Reagent Kit for library construction. Gene expression levels were quantified using RSEM software (http://deweylab.github.io/RSEM/). Principal component analysis (PCA) was performed on the gene expression data using R4.3.0 software to evaluate the overall quality of the RNA sequencing.

### Differential gene screening and pathway enrichment analysis

Differential gene expression(DEGs) analysis was performed using the “limma” R package, with a threshold set at p-values < 0.05 and |logFC| ≥ 1.5.Gene Ontology (GO) enrichment analysis was conducted using the GO database (https://github.com/bbuchfink/diamond) [14], which categorizes genes based on Molecular Function (MF), Cellular Component (CC), and Biological Process (BP). DEGs were annotated according to these categories. Pathway enrichment analysis was performed using the Kyoto Encyclopedia of Genes and Genomes (KEGG) database (http://www.genome.jp/kegg/) [15], which categorizes genes based on the biological pathways they are involved in. KEGG annotation was performed to identify relevant pathways associated with the DEGs.

### Identification and enrichment analysis of DE-PCDs in *S. aureus* pneumonia

A comprehensive collection of PCD-related genes was systematically curated from seven authoritative biomedical resources: peer-reviewed literature [16], the KEGG pathway database [17], the GeneCards gene annotation platform [18], molecular feature compendia [19], the Reactome pathway repository [20], the Autophagy Database (http://www.autophagy.lu/index.html), and the FerrDB ferroptosis database (http://zhounan.org/ferrdb). This integrated dataset spans 13 mechanistically distinct PCD subtypes, including apoptosis (136 genes), ferroptosis (382 genes), autophagy (222 genes), necroptosis (159 genes), lysosome-dependent cell death (255 genes), pyroptosis (33 genes), oxeiptosis (26 genes), netosis (17 genes), cuprotosis (14 genes), entotic cell death (15 genes), alkaliptosis (7 genes), parthanatos (9 genes), and disulfidptosis (4 genes). Subsequential analysis identified DE-PCDs through intersection of transcriptomic data with the curated PCD gene set, with results visualized via Venn diagrams using the R package “venn”.

### Identification of DE-PCDs in patients with *S. aureus* pneumonia

The transcriptomic dataset GSE30119 was retrieved from the Gene Expression Omnibus (GEO) database, comprising 99 peripheral blood samples from Staphylococcus aureus pneumonia patients and 44 healthy controls. Probe-level expression data were annotated to gene symbols using Perl-based pipelines with platform-specific probe-gene mapping files. Differential expression analysis was conducted via the “limma” R package. Statistically significant differentially expressed genes (DEGs) were defined by adjusted p-values < 0.05 and |logFC| ≥ 1.5. Subsequent characterization of DE-PCDs leveraged the same analytical framework, with expression patterns visualized through customized boxplots generated by the “ggpubr” package.

### Machine learning-based identification of PCD-related biomarkers

Machine learning methods Support Vector Machine-Recursive Feature Elimination (SVM-RFE) and Least Absolute Shrinkage and Selection Operator (LASSO) were used to screen for the characteristic DE-PCDs of *S. aureus* pneumonia. SVM-RFE was selected for its capability to recursively eliminate low-contribution features while preserving high-dimensional data relationships, with parameters optimized via 10-fold cross-validation (radial basis kernel; cost = 1). LASSO regression was chosen to handle multicollinearity among DE-PCDs through L1-penalized feature selection, where the optimal lambda (λ = 0.021) was determined by minimum binomial deviance in 1000 bootstrap iterations. The SVM module was constructed using the R package e1071 [21], while LASSO regression was implemented with the “glmnet” R package [22,23]. To prevent overfitting, both models employed stratified cross-validation during training, and final features were validated in independent test sets. The DE-PCDs identified by both methods were intersected using the “Venn” R package. A violin plot was used to display the expression patterns of these genes in the dataset. The diagnostic value of the identified DE-PCDs was assessed using ROC curves [24], with *p* < 0.05 considered statistically significant.

### Construction of nomogram model based on DE-PCDs

The “glmnet” package was used to further identify model DE-PCDs [25]. Nomograms were constructed based on clinical features and the model DE-PCDs using the “rms” R package. Calibration curves were used to evaluate the accuracy of the nomogram [26]. The clinical application value of the nomogram was analyzed by decision curve.

### Analysis of peripheral blood immune microenvironment

To investigate the immune microenvironment in the peripheral blood of *S. aureus* pneumonia patients, the CIBERSORT method was employed to assess the infiltration and proportions of 22 immune cell subsets [27]. Pearson correlation analysis was performed to evaluate the relationship between the characteristic DE-PCDs and immune cells in the peripheral blood.

### Quantitative PCR (qPCR)

Total RNA was isolated using TRIzol™ reagent (Invitrogen) according to established protocols [28]. cDNA synthesis was performed with the PrimeScript™ RT Reagent Kit (Takara Bio; cat# RR037A). Quantitative PCR assays were conducted using TB Green® Premix Ex Taq™ II (Takara Bio; cat# RR820A) on an Applied Biosystems 7500 Fast Real-Time PCR System.Primer sequences for all genes analyzed by qPCR are provided in S1 Table. Thermal cycling parameters were: 95°C for 3 min; 40 cycles of 95°C for 5 s and 60°C for 34 s [29]. Gene expression was quantified via the 2 − ΔΔCT method normalized to β-actin. Due to limited tissue availability, qRT-PCR validation for different genes was performed using independent sets of biological samples.All primers (designed and synthesized by Gensys Biotechnology) demonstrated single-band specificity confirmed by 2% agarose gel electrophoresis [30].

### Prediction of DE-PCDs related drugs and its molecular docking for targeting model genes

Potential drug targets interacting with the identified DE-PCDs were predicted using the DSigDB online database (http://tanlab.ucdenver.edu/DSigDB) [31]. These potential therapeutic agents may target DE-PCDs for the treatment of *S. aureus* pneumonia. Protein-small molecule interaction networks were constructed using Cytoscape to visualize the potential drug-target relationships. Subsequently, molecular docking analysis with was used to decrypt the interactions between these drugs and diagnostic markers. First, inhibition UDP-N-acetylmuramoyl-L-alanyl-D-glutamate-2,6-diaminopimelate ligase (MurE), as a key catalysis in cell wall synthesis of S. aureus, can cause inhibition of bacterial growth and lysis due to loss of osmotic resistance. The steric structure of *S. aureus* MurE (saMurE, PDB ID: 4C13) and methicillin-resistant *S. aureus* receptors (3wqu and 5bs3) were downloaded from the Protein Data Bank (https://www.rcsb.org/).

### Statistical analysis

Statistical analyses were performed using GraphPad Prism 9.0.0 software. The Shapiro-Wilk test was used to assess the normality of the data. The independent t-test was applied for comparisons between two groups, and data were presented as mean ± standard deviation (SD). For non-normally distributed data, the Mann-Whitney U test was used. A p-value < 0.05 was considered statistically significant.

## Results

### DEGs screening and functional enrichment

**Fig 1:**
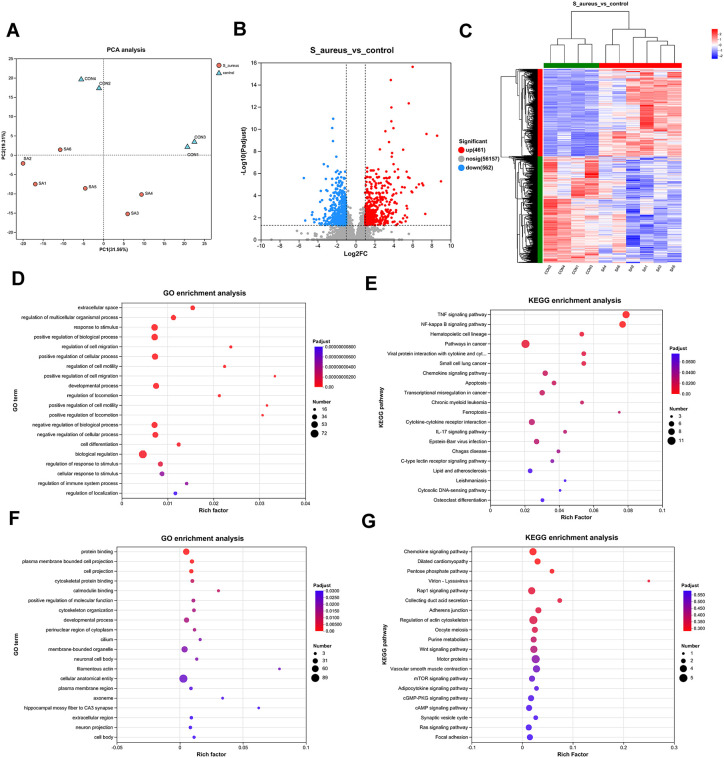
Identification and enrichment analysis of DEGs in *S. aureus* pneumonia. **(A)** PCA analysis of RNA-seq. **(B, C)** Volcano plot and heatmap of the DEGs from control and *S. aureus* groups. **(D, E)** GO and KEGG enrichment analyses based on the upregulate DEGs. **(F, G)** GO and KEGG enrichment analyses based on the downregulated DEGs.

PCA of transcriptomic profiles revealed distinct clustering patterns between *S. aureus* infected and control groups (Fig 1A), demonstrating satisfactory data quality and inter group discriminative capacity. Comparative analysis identified 1,536 DEGs with statistical significance (p < 0.05), comprising 674 upregulated and 862 downregulated transcripts (Figs 1B and 1C, S2 Table). GO analysis of upregulated DEGs demonstrated significant enrichment in regulatory processes, particularly biological regulation, cellular process modulation, and metabolic regulation (Fig 1D), all critical for host-pathogen interactions in pneumonia. KEGG pathway analysis further revealed predominant involvement of upregulated genes in critical inflammatory and programmed cell death pathways, including TNF signaling, NF-κB activation, apoptosis, and ferroptosis (Fig 1E). These coordinated transcriptional changes suggest *S. aureus* infection induces multifaceted activation of pro-inflammatory cascades and regulated cell death mechanisms in pulmonary tissues. In contrast, the downregulated DEGs were mainly linked to biological processes such as protein binding, plasma membrane-bound cell projections, and chemokine signaling, including the Rap1 and mTOR signaling pathways (Figs 1F and 1G), which potentially impairing host defense. Study had found weakened protein interactions reduce bacterial clearance, diminished pseudopodia hinder phagocytosis, and suppressed chemokine signaling may delay adaptive immune responses [32,33]. This aligns with reports that *S. aureus* secretes factors like CHIPS to disrupt chemokine function and evade immunity. Collectively, these findings suggest *S. aureus* infection modulates host defense through altered protein interactions and immune signaling.

### Screening and functional enrichment of PCD-associated DEGs

**Fig 2:**
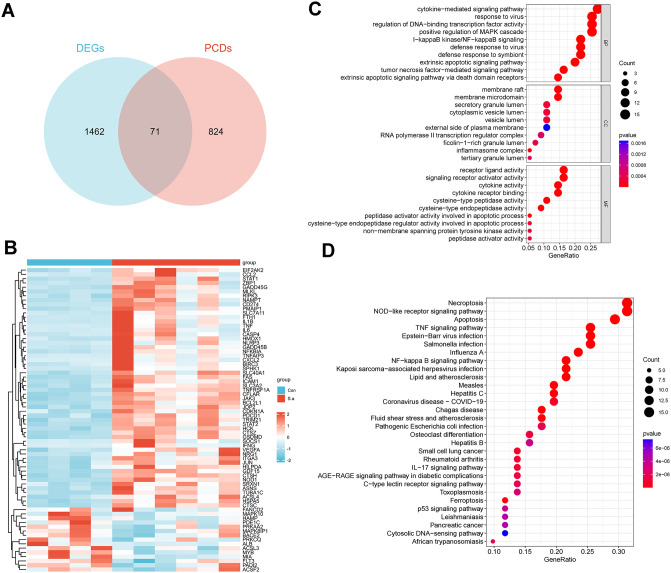
Screening and enrichment analysis of DE-PCDs. **(A)** Venn diagram of DE-PCDs. **(B)** Heatmap of the DE-PCDs. **(C, D)** GO and KEGG enrichment analyses based on the DE-PCDs.

Computational intersection analysis of 1,533 DEGs with 895 known DE-PCDs (Figs 2A and 2B, S3 Table). Systematic bioinformatics interrogation through integrated GO and KEGG functional enrichment analyses delineated the pathobiological significance of these DE-PCDs. GO analysis revealed marked enrichment in cytokine-mediated signaling pathways, particularly TNF receptor superfamily signaling, inflammasome assembly, and cytokine receptor activity (Fig 2C). KEGG pathway mapping demonstrated significant enrichment of DE-PCDs in necroptosis, apoptosis, NOD-like receptor signaling, and ferroptosis (Fig 2D). Collectively, these findings delineate a pathogenic paradigm wherein the coordinated activation of necroptosis, apoptosis, and ferroptosis synergistically drive pulmonary immunopathology during *S. aureus* infection.

### Cross-species validation of PCD gene signatures

**Fig 3:**
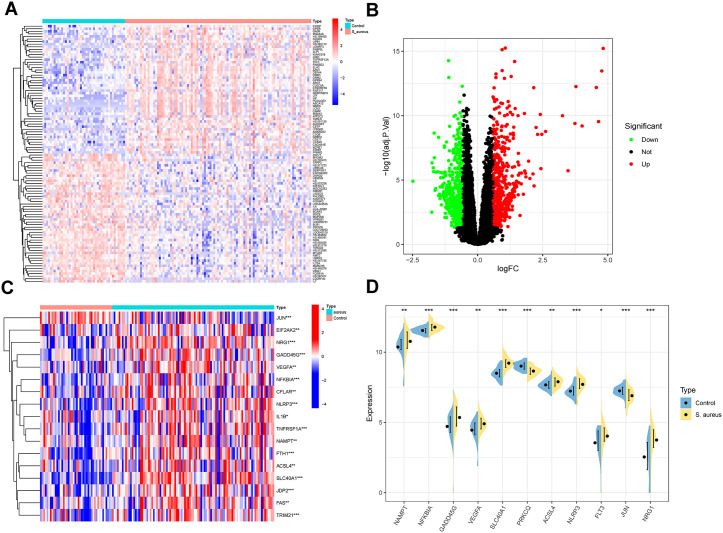
DE-PCDs in Peripheral Blood of Patients with S. aureus Pneumonia. **(A, B)** The heatmap and volcano plot of the DEGs from GSE30119. **(C, D)** DE-PCDs in the peripheral blood of S. aureus pneumonia patients compared to healthy controls (* P < 0.05, ** P < 0.01, *** P < 0.001).

To evaluate the clinical relevance of murine findings, we analyzed the publicly available GSE30119 dataset containing peripheral blood transcriptional profiles from *S. aureus* pneumonia patients. Comparative analysis identified 1,127 DEGs in patient samples compared to healthy controls (Figs 3A and B). Strikingly, 19 DE-PCDs identified in murine lung tissues exhibited consistent differential expression in human peripheral blood (Figs 3C and 3D). Notably, the DE-PCDs encompassed critical regulators of necroptosis, apoptosis, and ferroptosis pathways previously identified in murine models. This interspecies conservation suggests these 19 DE-PCDs may play critical regulatory roles in orchestrating programmed cell death during *S. aureus* pneumonia pathogenesis.

### Machine learning-based PCD diagnostic signature

**Fig 4:**
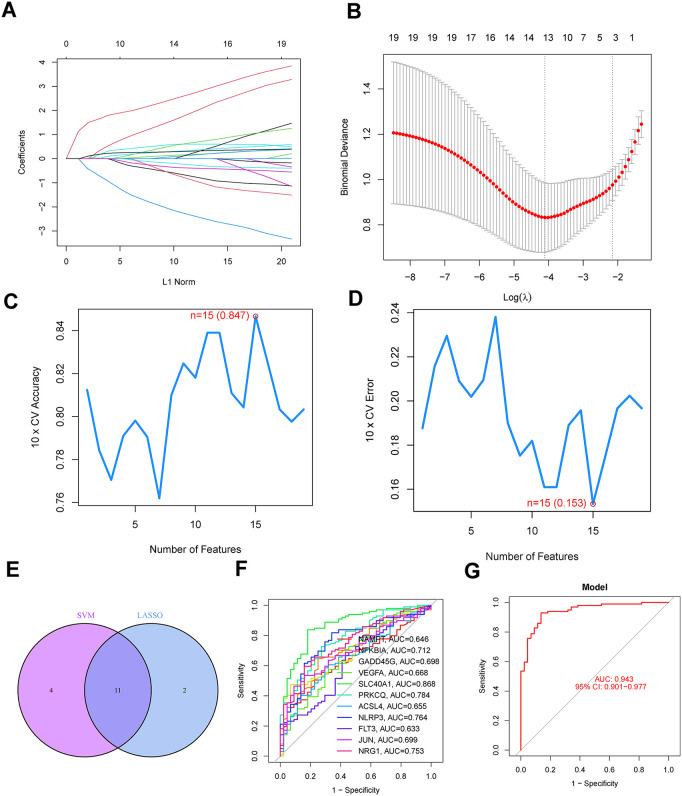
Machine Learning Screening of Characteristic DE-PCDs and Diagnostic Analysis. **(A)** Distribution of LASSO coefficients for DE-PCDs. **(B)** Ten fold cross-validation and optimal parameter (lambda)for DE-PCDs coefficients. **(C, D)** Establishment of characteristic DE-PCDs based on SVM-RFE algorithm (n = 15). **(E)** Venn diagram of intersection between LASSO and SVM-RFE methods; **(F)** ROC analysis of Characteristic DE-PCDs; **(G)** Diagnostic model based on Characteristic DE-PCDs.

To establish clinically actionable biomarkers, we implemented two feature selection algorithms on the DE-PCDs dataset. SVM-RFE identified 15 candidate biomarkers (Figs 4A and 4B,S4 Table). while LASSO regression independently selected 15 features (Figs 4C and 4D,S5 Table). Intersection analysis revealed 11 consensus biomarkers: NAMPT, NFKBIA, GADD45G, VEGFA, SLC40A1, PRKCQ, ACSL4, NLRP3, FLT3, JUN, and NRG1 (Fig 4E). Functional annotation delineated distinct pathway associations among the identified biomarkers, with NFKBIA and GADD45G governing apoptotic regulation, EIF2AK2 and NLRP3 mediating necroptotic signaling, NAMPT, PRKCQ, and NRG1 modulating autophagic processes, while VEGFA, SLC40A1, ACSL4, FLT3, and JUN orchestrated ferroptosis execution, collectively spanning four major programmed cell death modalities implicated in S. aureus pathogenesis. The logistic regression model incorporating these biomarkers demonstrated strong diagnostic accuracy (AUC = 0.943) in distinguishing S. aureus pneumonia cases (Figs 4F and 4G). This integrative computational framework, integrating dual-algorithm feature selection and multivariate regression, defines an eleven-gene molecular signature with demonstrated translational potential for differential diagnosis of *S. aureus* pneumonia through concurrent modulation of apoptosis, necroptosis, autophagy, and ferroptosis pathways.

### Diagnostic model construction

**Fig 5:**
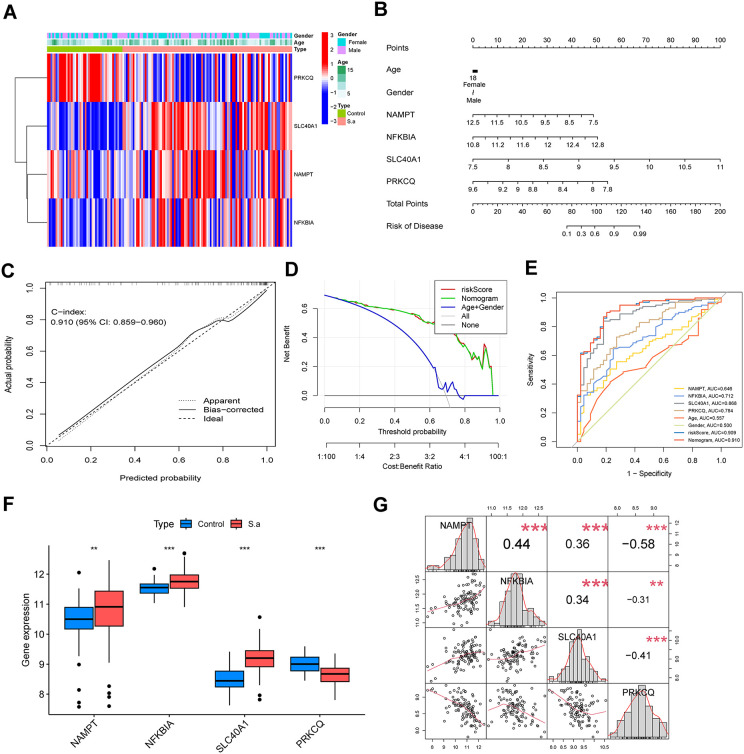
Construction of Diagnostic Model base on characteristic DE-PCDs. **(A)** Heatmap of model DE-PCDs. **(B)** The nomogram shows the prognostic value of model DE-PCDs, age and gender in *S. aureus* pneumonia patients. **(C)** The degree of similarity between the actual results and the predicted results of the model DE-PCDs related nomogram was assessed using the calibration curve. **(D)** ROC analysis base on model DE-PCDs and nomogram. **(E)** Decision curve analysis was used to evaluate the sensitivity and specificity of the model DE-PCDs related nomogram. **(F)** Boxplot shows the expression of model DE-PCDs in peripheral blood of *S. aureus* pneumonia patients. **(G)** Correlation heatmap of the model DE-PCDs expressed in *S. aureus* pneumonia, the red lines indicate the fitted curves. (* P < 0.05, ** P < 0.01, *** P < 0.001).

Employing the “glmnet” package, we refined the 11-gene signature to a core 4 model genes (NAMPT, NFKBIA, SLC40A1, PRKCQ) (Fig 5A). A multivariate nomogram integrating these biomarkers with demographic variables (age, sex) demonstrated excellent predictive accuracy (C-index = 0.910) through calibration curve analysis (Figs 5B and 5C). The Decision curve analysis (DCA) confirmed clinical utility across the full risk threshold spectrum (0–100%) (Fig 5D). Further validation of the diagnostic value of the model genes was conducted using a ROC curve. The composite diagnostic model demonstrated superior discriminative capacity (AUC = 0.910), significantly outperforming individual gene performance with SLC40A1 achieving the highest single-gene AUC of 0.868, followed by PRKCQ (0.784), NFKBIA (0.712), and NAMPT (0.646), collectively highlighting the synergistic predictive power of multimodal pathway integration (Fig 5E). The nomogram exhibited strong diagnostic potential, with SLC40A1 (AUC = 0.868) and PRKCQ (AUC = 0.784) serving as primary predictive contributors for *S. aureus* pneumonia. Multivariate analysis revealed no significant improvement in predictive accuracy when incorporating age or gender. Transcriptional profiling confirmed marked dysregulation of all four model genes (NAMPT, NFKBIA, SLC40A1, PRKCQ) in pneumonia cases versus controls (p < 0.001, Fig 5F). Intergene correlation analysis identified a significant negative association between NAMPT and PRKCQ expression (Fig 5G), suggesting counterregulatory interactions within the diagnostic signature.

### DE-PCDs mediated immune infiltration analysis

**Fig 6:**
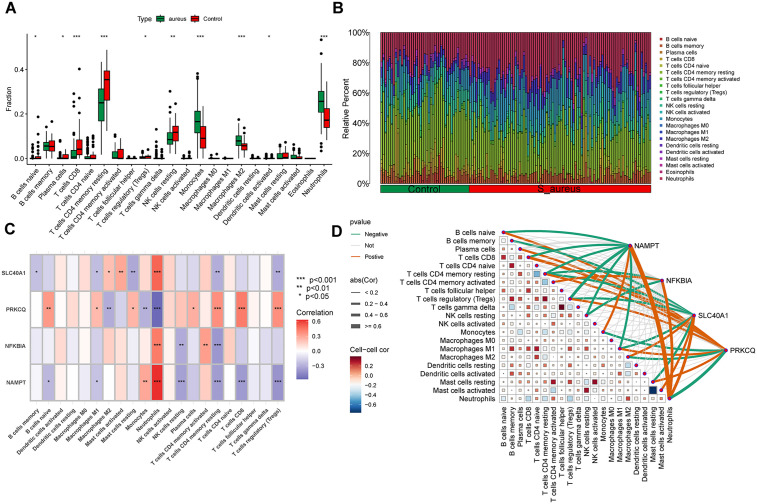
Correlation analysis between model DE-PCDs and immune microenvironment. **(A, B)** Expression differences of 22 distinct immune infiltrating cells between *S. aureus* pneumonia and Control groups **(C)** The correlation heatmap of the model DE-PCDs and immune infiltrating cells in *S. aureus* pneumonia. **(D)** The correlation network of the model DE-PCDs and immune infiltrating cells, the orange line represents a positive correlation and the green line represents a negative correlation. (* P < 0.05, ** P < 0.01, *** P < 0.001).

The immune microenvironment plays a pivotal role in the pathogenesis of *S. aureus* pneumonia [1,34]. To delineate the immunological relevance of the identified DE-PCDs, we systematically characterized the immune landscape of peripheral blood in *S. aureus* pneumonia patients using CIBERSORT deconvolution, followed by Pearson correlation analysis between DE-PCDs and immune cell fractions. Comparative analysis revealed significant immunological remodeling in the *S. aureus* infection cohort, marked by elevated proportions of monocytes, M2-polarized macrophages, neutrophils, and activated dendritic cells relative to controls. Conversely, substantial reductions were observed in naive B cells, CD8 + T lymphocytes, regulatory T cells, and resting NK cells (Figs 6A and 6B,S6 Table). Notably, Pearson correlation mapping demonstrated distinct DE-PCDs–immune cell interactions. Specifically, SLC40A1, NFKBIΑ1 and NAMPT exhibited strong positive correlations with neutrophil infiltration. In contrast, while PRKCQ inversely correlated with neutrophils, activated CD4 + T cells, CD8 + T effectors, and Tregs (Figs 6C and 6D). These findings suggest that DE-PCDs may contribute to immune microenvironment alterations in *S. aureus* pneumonia, warranting further investigation into their therapeutic potential.

### Drug target prediction and molecular docking

**Fig 7:**
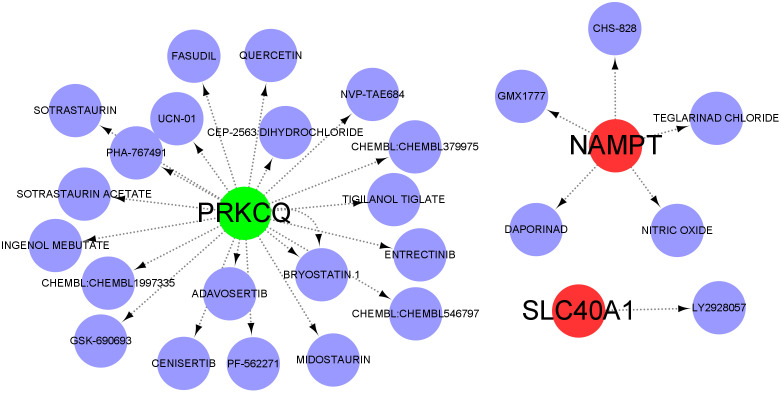
Drug target prediction of model DE-PCDs.

**Fig 8:**
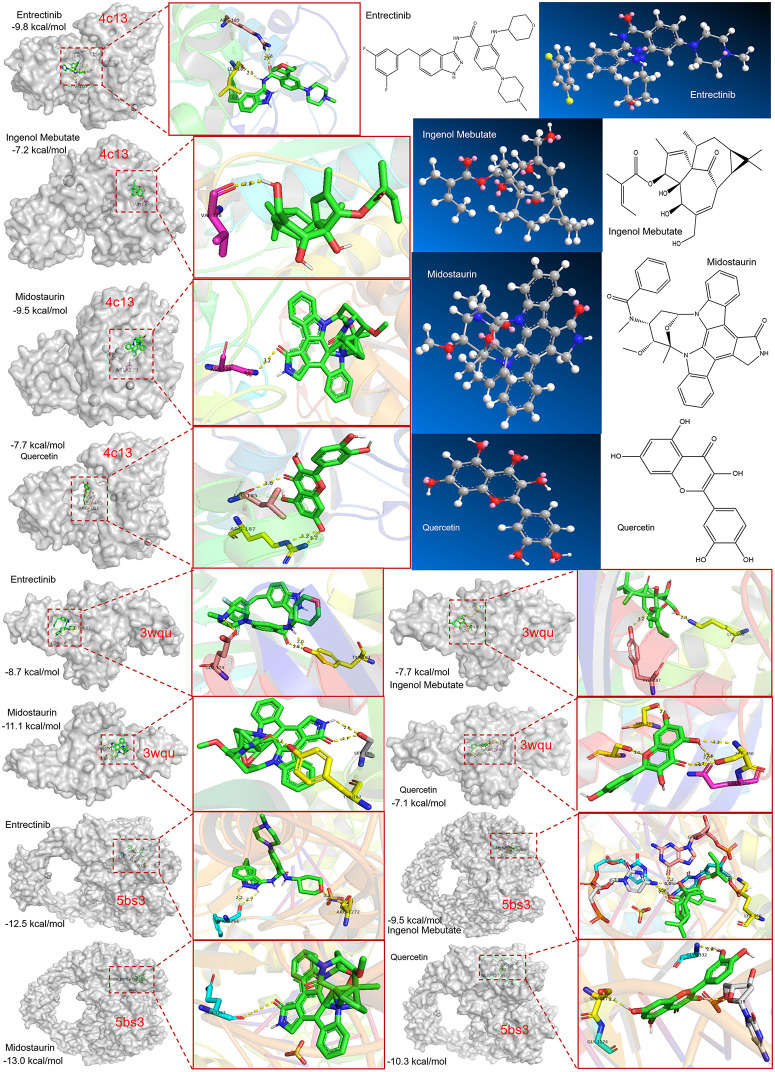
Intermolecular interactions of 4 approved potential small molecular drug with the key protein of *S. aureus* infection.

Anti-disease small molecule compounds were predicted by DSigDB database to explore potential therapeutic interventions for *S. aureus* pneumonia. Four model DE-PCDs were selected for drug target prediction. As a result, 26 target drugs with potential therapeutic effects were predicted in Fig 7 and S7 Table. Among them, 20 small‑molecule drugs were found to correlate with PRKCQ and five target drugs (CHS-828, GMX1777, TEGLARINAD CHLORIDE, NITIRIC OXIDE and DAPORINAD) were associated with NAMPT. There is a drug (LY2928057) related to SLC40A1. The predicted small molecules suggesting that these DE-PCDs may serve as potential inhibitors for *S. aureus* pneumonia progression. These findings imply that the model DE-PCDs could be targeted by specific drugs to potentially alleviate the disease or modulate the immune response involved in S. aureus pneumonia. Subsequently, the molecular docking simulation was further studied to clarify the intermolecular interaction between the four approved drugs (Entrectinib, Ingenol mebutate, Midostaurin and Querectin) and the target proteins of *S. aureus*. As shown in Fig 8, four drug-protein interactions with the corresponding primary proteins in *S. aureus* showed higher binding affinity energy (≤− 7 kcal/mol). Thus, these data showed that 4 drugs had a higher stable binding to proteins, resulting in the potential for higher therapeutic effects. Furthermore, two binding residues (Leu185 and Arg187) were mutual intermolecular interaction sites in the structure of 4c13. A structural domain containing Tyr187, Ser191, Lys198, Sre350, Gln351, Ser364, Ser367, Glu374 residues can be observed at the sequence of 3wqu. Residues Lys466, Ser1173, Gly1174, Phe1266, Gln1267, Gly1332, C3, C4 and C19 are involved in the active pocket of 5bs3.

### Experimental validation of DE-PCDs signatures

**Fig 9:**
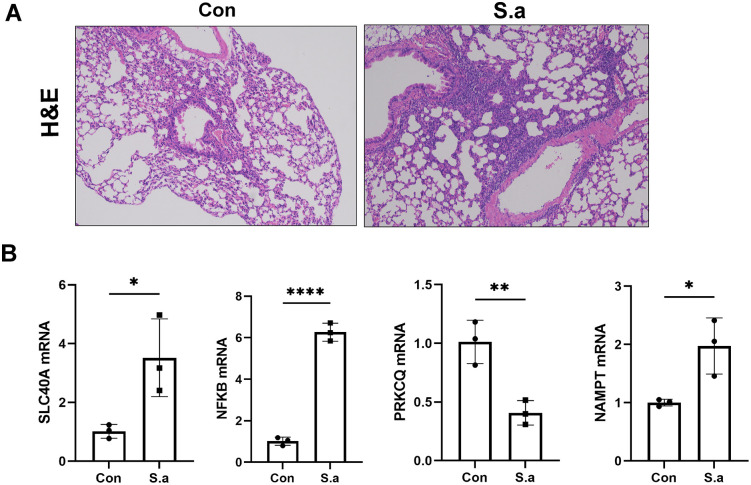
Validation of the model DE-PCDs in *S.aureus* pneumonia mice. **(A)** HE staining analysis of lung tissue. **(B)** Changes of mRNA expression levels of model DE-PCDs in mice sample. (* P < 0.05, ** P < 0.01, *** P < 0.001, **** P < 0.0001).

To validate the in vivo expression patterns of model DE-PCDs, we established a murine *S. aureus* pneumonia model via intranasal instillation of bacterial suspension. Histopathological examination using hematoxylin-eosin (HE) staining demonstrated robust peribronchial inflammatory infiltrates in infected mice (*S.a* group), confirming successful model establishment (Fig 9A). Consistent with our bioinformatics predictions, qRT-PCR analysis revealed significant upregulation of SLC40A1, NFKBIΑ1, and NAMPT in lung tissues of *S. aureus* infected mice compared to sham controls (P < 0.05). In contrast, PRKCQ expression was markedly downregulated in the infection group (Fig 9B).The raw Ct values for the qPCR validation experiments are provided in S8 Table.These orthogonal validation results corroborate the relevance of the identified DE-PCDs in *S. aureus* pneumonia pathogenesis.

## Discussion

*Staphylococcus aureus* pneumonia remains a formidable clinical challenge due to escalating antibiotic resistance and poorly understood host-pathogen dynamics. While programmed cell death (PCD) pathways are recognized as critical modulators of infection outcomes, their coordinated regulation in *S. aureus* pneumonia pathogenesis, particularly as dual-purpose theranostic targets has remained elusive [5]. Understanding the molecular mechanisms underlying *S. aureus* pneumonia, particularly the role of specific genes and cell death pathways, is crucial for the development of effective diagnostic and therapeutic interventions.

Through cross-species transcriptomic analysis, we identified 71 DE-PCDs that converge on four evolutionarily conserved cell death modalities: apoptosis, necroptosis, autophagy, and ferroptosis. Notably, pathway enrichment revealed *S. aureus* infection induces concurrent activation of both inflammatory (necroptosis/ferroptosis) and immunosuppressive (apoptosis/autophagy) PCD programs. This paradoxical regulation aligns with emerging paradigms in microbial pathogenesis, wherein pathogens exploit host cell death machinery to simultaneously promote tissue invasion while evading immune surveillance [35–37]. Specifically, previous studies have demonstrated that *S. aureus* induced apoptosis/autophagy may facilitate bacterial persistence by eliminating effector immune cells (e.g., alveolar macrophages) [38,39]. On the other hand, concurrent activation of necroptosis/ferroptosis may drives inflammatory lung injury through RIPK1/MLKL signaling and iron-dependent lipid peroxidation [40,41]. This mechanistic duality was further corroborated by immune profiling showing elevated immunosuppressive M2 macrophages and neutrophils cell populations particularly susceptible to PCD-mediated regulation. Strikingly, neutrophil infiltration exhibited the strongest correlation with our biomarker panel (NAMPT: r = 0.68; SLC40A1: r = 0.62), suggesting *S. aureus* may manipulate granulocyte death pathways to optimize infection outcomes through either delayed apoptosis (intracellular persistence) or accelerated necroptosis (tissue damage).

Building upon these mechanistic insights, our machine learning identified a four-gene diagnostic signature (NAMPT, NFKBIA, SLC40A1, PRKCQ) with superior predictive accuracy (AUC = 0.92). The biological plausibility of this panel is supported by their collective involvement in PCD-immune regulatory networks: NAMPT may act as a metabolic checkpoint in inflammatory cell death via NAD + -mediated inflammasome activation [42]. LC40A1, a critical iron transporter, suggests ferroptosis signaling could influence bacterial persistence through iron homeostasis dysregulation [43,44], while PRKCQ was found to be potential involvement in ferroptosis regulation, particularly through oxidative stress and lipid metabolism pathways relevant to infectious diseases [45]. Notably, NFKBIA (encoding IκBα) shows infection-dependent upregulation in SARS-CoV-2 infections [46], and extracellular NCOA4 exacerbates inflammatory responses through NF-κB activation in sepsis [47]. The functional synergy across metabolic, metal ion homeostasis, and immunoregulatory pathways underscores the network-level significance of this biomarker combination. Our study provides a systems-level perspective on PCD interactions, complementing conventional single-pathway analyses. Small-molecule compound screening predicted 20 candidates with potential binding affinity for hub genes, including four agents (e.g., quercetin and midostaurin) showing in silico binding capability to both host PCD regulators and *S. aureus* virulence proteins. Molecular docking simulations revealed stable interactions between quercetin and bacterial quorum-sensing components (e.g., AgrA), suggesting these compounds may dually modulate host-pathogen interactions.

While prior investigations have delineated individual programmed cell death pathways in bacterial infections, this study systematically characterizes the co-dysregulation of four PCD modalities (apoptosis, necroptosis, autophagy, ferroptosis) in *S. aureus* pneumonia through integrated transcriptomic profiling. By intersecting murine model data with human clinical datasets, we reveal conserved patterns of PCD gene interactions that correlate with disease progression, including coordinated activation of inflammatory and immunosuppressive death programs. This systems-level analysis extends beyond single-pathway observations, proposing testable hypotheses about how bacterial pathogens may exploit interconnected cell death networks to subvert host defenses.

However, several limitations should be acknowledged. Firstly, while the murine model provides valuable insights, it may not fully recapitulate the complexity of human disease, particularly in terms of immune responses and genetic variability. Additionally, the reliance on transcriptomic data and machine learning algorithms, while powerful, introduces potential biases and limitations in gene selection and pathway enrichment analyses. The validation of identified genes through qPCR, while essential, may not encompass all relevant biological contexts, potentially overlooking critical interactions within the immune microenvironment. Moreover, the predictive nature of the ROC model necessitates cautious interpretation, as it may not account for all confounding factors present in clinical settings. Future studies should aim to integrate multi-omics approaches and clinical data to enhance the translational relevance of these findings.

## Conclusion

This study integrates transcriptomic data analysis with machine learning algorithms to first reveal the crosstalk regulatory features of multiple PCD pathways in *S. aureus* pneumonia. We identified key PCD regulators with diagnostic and prognostic potential and screened potential therapeutic compounds, providing novel biomarkers and actionable intervention targets for precision management of *S. aureus* pneumonia. While limited by human sample validation needs, future work will employ CRISPR models to mechanistically dissect how key genes coordinate apoptosis, necroptosis, and ferroptosis, advancing targeted intervention strategies.

## Supporting information

S1 Table Primer sequences used for qPCR. (DOCX)

S2 Table Differentially expressed genes between control and S. aureus–infected groups. (XLSX)

S3 Table Differentially expressed programmed cell death–related genes (DE-PCDs). (XLSX)

S4 Table Feature genes identified by the support vector machine (SVM) model. (TXT)

S5 Table Feature genes identified by the LASSO regression model. (TXT)

S6 Table Immune cell infiltration results estimated by CIBERSORT. (TXT)

S7 Table Predicted gene–drug interaction results. (TXT)

S8 Table Raw Ct values for qPCR validation experiments. (XLSX)
