# Supplementary material for: Cell death-related gene signatures as dual-function biomarkers: Early diagnosis and therapeutic targeting in Staphylococcus aureus pneumonia
Source: PLoS One. 2026 Jan 20;21(1):e0339560. doi: 10.1371/journal.pone.0339560 (PMC12818594; doi:10.1371/journal.pone.0339560)
Supplement: S1 Table — (DOCX) [file pone.0339560.s001.docx]

**Table S1.** Primer Sequences in RT-qPCR

| **Gene Name** | **Primer Sequence (5’-3’)** |
| --- | --- |
| Mice-NAMPT-F | GATACTGTGGCGGGAATTGCT |
| Mice-NAMPT-R | CTCATGGTCTTTCCCCCAAGC |
| Mice-NFKBIA-F | CCACCAACTACAATGGCCACAC |
| Mice-NFKBIA-R | CAGCACCCAAAGTCACCAAGT |
| Mice-SLC40A1-F | ACAGGAGCAGATTAGCAGACAT |
| Mice-SLC40A1-R | GGACACCAAATTCCAACCGGA |
| Mice-PRKCQ-F | TTGAAAGCACCCAACAGGCTC |
| Mice-PRKCQ-R | TTTCCCAGGTGTTGGTACGC |
| Mice-GAPDH-F | CATCACTGCCACCCAGAAGACTG |
| Mice-GAPDH-R | ATGCCAGTGAGCTTCCCGTTCAG |
